# Hepatitis B virus X protein is capable of down-regulating protein level of host antiviral protein APOBEC3G

**DOI:** 10.1038/srep40783

**Published:** 2017-01-18

**Authors:** Ruidong Chen, Xue Zhao, Yongxiang Wang, Youhua Xie, Jing Liu

**Affiliations:** 1 Key Laboratory of Medical Molecular Virology (MOE/MOH) and Institutes of Biomedical Sciences, Shanghai Medical College, Fudan University, Shanghai 200032, People’s Republic of China; 2 Microbiology Laboratory, Shanghai Municipal Center for Disease Control and Prevention, Shanghai 200336, People’s Republic of China

## Abstract

The apolipoprotein B mRNA editing catalytic polypeptide-like (APOBEC) family proteins bind RNA and single-stranded DNA, and create C-to-U base modifications through cytidine deaminase activity. APOBEC3G restricts human immunodeficiency virus 1 (HIV-1) infection by creating hypermutations in proviral DNA, while HIV-1-encoded vif protein antagonizes such restriction by targeting APOBEC3G for degradation. APOBEC3G also inhibits hepatitis B virus (HBV): APOBEC3G co-expression inhibits HBV replication and evidences exist indicating APOBEC3G-mediated HBV hypermutations in patients. HBV encodes a small non-structural X protein (HBx) with a recognized activating effect on HBV life cycle. In this work, we report the discovery that HBx selectively and dose-dependently decreases the protein level of co-expressed APOBEC3G in transfected Huh-7 cells. The effect was shown to take place post-translationally, but does not rely on protein degradation via proteasome or lysosome. Further work demonstrated that intracellular APOBEC3G is normally exported via exosome secretion and inhibition of exosome biogenesis causes retention of intracellular APOBEC3G. Finally, HBx co-expression specifically enhanced externalization of APOBEC3G via exosomes, resulting in decrease of intracellular APOBEC3G protein level. These data suggest the possibility that in addition to other mechanisms, HBx-mediated activation of HBV might also involve antagonizing of intracellular restriction factor APOBEC3G through promotion of its export.

Hepatitis B virus (HBV) is the type member of the *Hepadnaviridae* family of enveloped pararetroviruses and naturally infects human beings with a nearly exclusive tropism for hepatocytes^1^,^2^. Infection by HBV causes subclinical or symptomatic acute hepatitis, and acute hepatitis may develop into chronic HBV infection, which generally persists for life and has been proven to be associated with higher risk of liver fibrosis, cirrhosis and hepatocellular carcinoma (HCC)^3^. World Health Organization estimated that worldwide, HBV chronically infects ~240 million people and contributes to about 686, 000 deaths annually^4^.

HBV genome harbours 4 overlapping open reading frames (ORF) and expresses 7 viral proteins through the use of different starting codons^1^. HBV X (HBx) is a small protein (154 amino acid residues) that is encoded by an ORF conserved in all mammalian hepadnaviruses. HBx is not considered to be a viral structural protein and numerous studies over the decades have shown that HBx plays a myriad of functions in regulating viral life cycle, host-virus interactions, as well as HBV-related HCC^5^,^6^,^7^,^8^. Most notably, HBx acts to stimulate the activities of HBV promoters and enhancers: obliteration of functional HBx ORF results in prominently reduced viral transcription and replication *in vitro*, which could be rescued by supplementing HBx expression *in trans*^9^,^10^. Infection experiments *in vivo* further confirms that HBx is essential for initiating and maintaining HBV life cycle^7^. HBx apparently does not bind to HBV DNA directly, and mechanistic studies of HBx-mediated augmentation of HBV have generally focused on interactions between HBx and cellular proteins/processes, and multiple targets of HBx have been identified^11^.

The APOBEC (apolipoprotein B mRNA editing catalytic polypeptide-like) family consists of evolutionarily and structurally related proteins that bind RNA and single-stranded DNA (ssDNA) and, through a conserved zinc-dependent cytidine deaminase activity, create C to U base modifications on substrate ssDNA and, for some members, also RNA^12^. Human APOBECs include APOBEC1, AID (activation-induced deaminase), APOBEC2, APOBEC3 subfamily containing 7 chromosomally clustered members (A, B, C, D/DE, F, G and H) and recently described APOBEC4. In addition to roles played in vital physiological processes such as somatic hypermutation and class-switch recombination in activated B cells, APOBEC-mediated editing has been shown to serve as an important part of innate immunity^12^,^13^. The most significant example is the discovery that APOBEC3G (A3G) and some other APOBEC3 subfamily proteins restrict human immunodeficiency virus 1 (HIV-1) infection by heavily editing viral ssDNA during reverse transcription resulting in hypermutated proviral DNA^14^. On the other hand, the vif protein encoded by HIV-1 specifically binds multiple A3 proteins including A3G, and targets the later for ubiquitination and proteasome degradation, thus antagonizing A3-mediated restriction^15^. In the context of HBV, it has been shown that A3G potently restricts HBV replication *in vitro*, in a manner that is apparently independent of its deaminase activity^16^,^17^,^18^. However, other *in vitro* and *in vivo* evidences suggested that A3 deaminase-mediated editing and subsequent hypermutations can and do affect HBV^19^,^20^,^21^,^22^. Regardless of the involvement of cytidine deaminase activity, A3G has been generally accepted as a restricting factor of HBV. Unlike HIV-1, no HBV-encoded protein has been shown to regulate A3G.

In this work, we report the discovery that HBx is capable of selectively and specifically down-regulating intracellular protein level of co-expressed A3G. Our data indicate that such down-regulation of A3G by HBx is most likely effected on already synthesized A3G proteins at the post-translational level, but does not seem to involve A3G protein degradation through proteasome or lysosome. Exosomes are small (~30–100 nm in diameter) membrane vesicles released by most cell types through fusion between intracellular multivesicular bodies (MVBs) and plasma membrane^23^. We provide evidences showing that intracellular A3G is normally exported in exosomes and such externalization of A3G is enhanced by HBx, which correlates with HBx-mediated down-regulation of intracellular A3G.

## Results

### HBx selectively decreases the intracellular protein level of APOBEC3G

**Figure 1:**
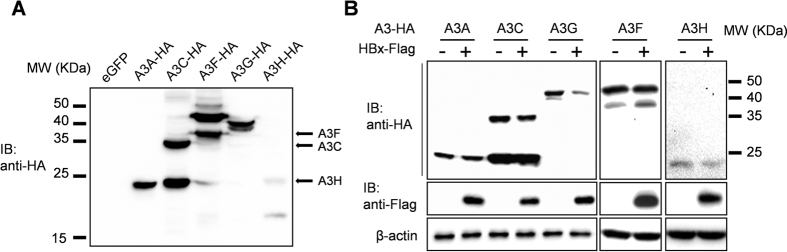
HBx selectively decreases A3G protein level. (**A**) Confirmation of expression of selected APOBEC3 (A3) family members. Huh-7 cells were transfected with expression plasmids encoding HA-tagged A3 proteins or eGFP control plasmid as indicated. Cells were harvested 48 hours post transfection and cell lysates were analyzed in parallel using immunoblot. Arrows indicate positions of full-length A3C, A3F and A3H. (**B**) Effects of HBx co-transfection on A3 protein levels. Huh-7 cells were transfected with indicated A3 member plasmids with or without HBx expression plasmid. Cells were harvested 48 hours post transfection. A3 proteins, HBx and β-actin were detected using antibodies as indicated.

In order to explore possible effects of HBx on APOBEC3 (A3) family members, expression plasmids encoding HA-tagged A3A, A3C, A3F, A3G and A3H under the control of cytomegalovirus (CMV) promoter were constructed and transfected into Huh-7 cells and expression of corresponding A3 proteins was confirmed in immunoblot using HA antibody (Fig. 1A). Plasmid expressing Flag-tagged HBx protein driven also by CMV promoter was then co-transfected with the A3 member expression plasmids. Immunoblot using Flag and HA antibodies revealed that protein levels of A3A, A3C, A3F and A3H displayed minimal differences with or without HBx co-expression, whereas A3G protein level in cells co-transfected with HBx was markedly lower compared to cells co-transfected with vector control (Fig. 1B). Apparently, among the members of A3 family tested here, only A3G is selectively affected by HBx.

When a mutant HBV genome with HBx ORF nulled (HBV-X^−^) was transfected into Huh-7 cells with or without co-transfected A3G and/or HBx, marked repression of viral replication could be observed in A3G co-transfected cells (Supplementary Fig. S1), reproducing previously reported results in the literature^17^,^24^. In cells co-transfected with both A3G and HBx, A3G level was expectedly lower compared to cells co-transfected with A3G alone, and HBV replication was also partially rescued (Supplementary Fig. S1). Although these data suggest that HBx-associated decrease in A3G level correlates with attenuated A3G restriction of HBV, as HBx itself markedly activates HBV-X^-^ replication in the absence of co-transfected A3G, distinguishing A3G-related and A3G-unrelated effects on replication in A3G and HBx co-transfected cells is difficult.

### HBx dose-dependently decreases APOBEC3G protein level

**Figure 2:**
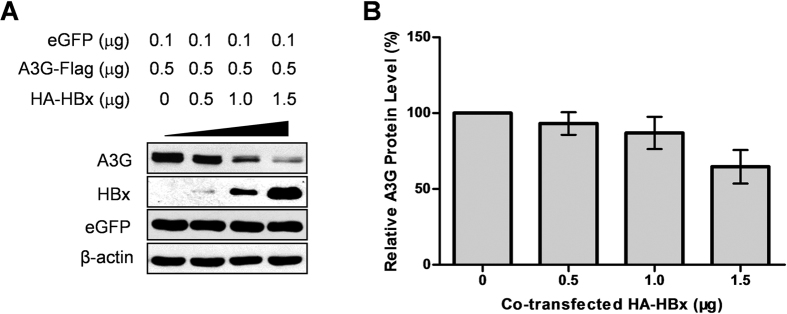
HBx dose-dependently decreases A3G protein level. (**A**) Huh-7 cells were transfected with A3G and increasing amounts of HBx as indicated. eGFP expression plasmid was used as transfection control. Cells were harvested 48 hours post transfection and cell lysates were analyzed in parallel in immunoblot. (**B**) Relative quantities of A3G in cell lysates as shown in (**A**) were estimated using densitometry scanning and normalized to eGFP quantities, taking values without HBx co-transfection as 1 (100%). Means and SD calculated from 3 independent experiments are shown.

To re-confirm the specificity of HBx’s effects on A3G protein level, the same amount of plasmid expressing Flag-tagged A3G was co-transfected with increasing amounts of HA-tagged HBx expression plasmid. Immunoblot data showed that higher amounts of transfected HBx-expressing plasmid resulted in more prominent reduction in A3G protein level, while no effect on protein level of eGFP expressed from co-transfected control plasmid was observed (Fig. 2A). Such dose-dependent decrease of A3G protein level by HBx was reproducible and calculation using densitometry scan data from multiple experiments indicated an average reduction of A3G protein level by ~5% when A3G and HBx were co-transfected at 1:1 ratio, by ~10% at 1:2 ratio and by ~35% at 1:3 ratio (Fig. 2B). It is therefore clear that HBx exerts selective and specific effects on the protein level of A3G.

### HBx decreases APOBEC3G protein level post-translationally

**Figure 3:**
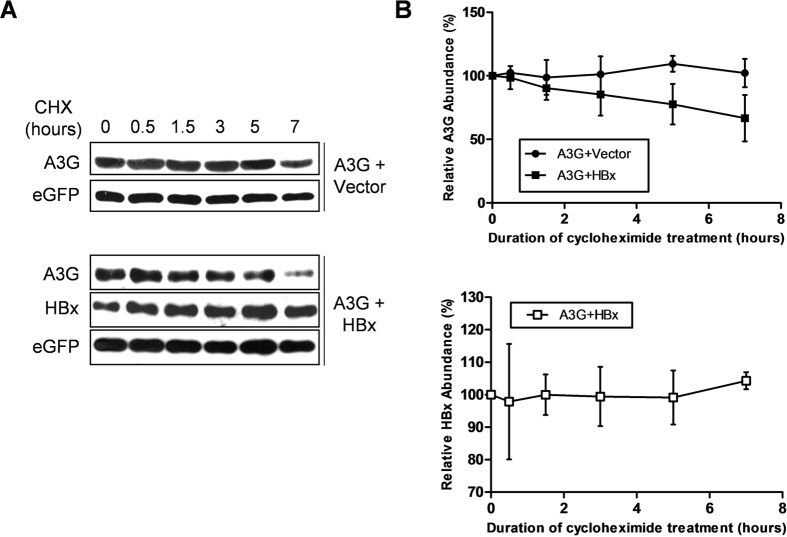
HBx decreases A3G protein level post-translationally. (**A**) Time-course study of A3G protein level following cycloheximide (CHX) treatment. Huh-7 cells transfected with A3G and eGFP expression plasmids, with or without HBx plasmid, were treated with CHX at 48 hours post transfection. Cells were collected at indicated time points and analyzed in parallel using immunoblot. (**B**) Relative quantities of A3G (top) and HBx (bottom) in cell lysates as shown in (**A**) were estimated using densitometry scanning and normalized against eGFP quantities, taking values at start of CHX treatment as 1 (100%). Means and SD calculated from 3 independent experiments are shown.

Since expression of HBx and A3G were both driven by CMV promoter in co-transfection assays, HBx is most likely to be down-regulating A3G protein at the post-transcriptional level. When transfected cells were treated with cycloheximide (CHX) to inhibit protein synthesis, intracellular A3G protein level displayed a gradual and marked decrease over then 7 hours of treatment when HBx was co-transfected, whereas in the absence of co-transfected HBx, there was only marginal decrease of A3G protein level towards the end of 7 hour treatment (Fig. 3A,B, top). In contrast, protein level of eGFP, used as transfection efficiency control, showed no notable change during CHX treatment, with or without HBx co-expression (Fig. 3A). Protein level of HBx did not change remarkably in response to CHX treatment either (Fig. 3B, bottom). These data indicated that HBx protein specifically and post-translationally decreases A3G protein level, without having to rely on affecting A3G mRNA transcription or translation. On the other hand, it cannot be ruled out that HBx might exert additional effects that interfere with transcription or translation, thus also contributing to the downregulation of A3G protein level.

### Degradation of APOBEC3G protein is not affected by HBx

**Figure 4:**
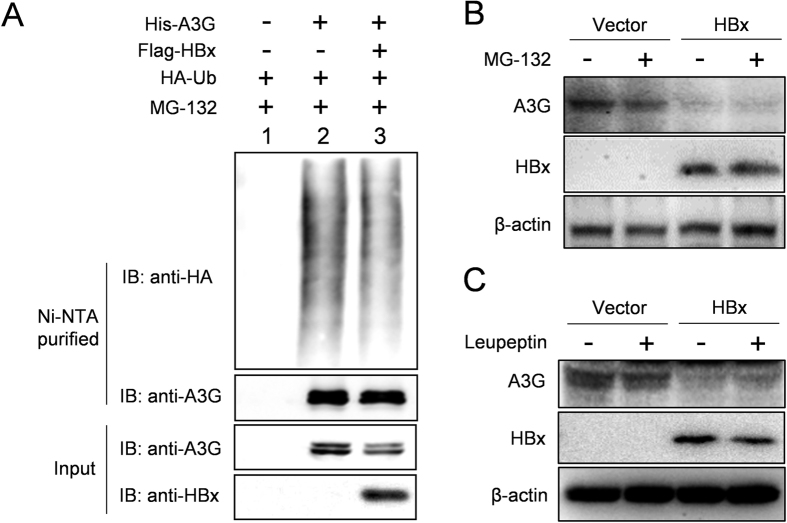
Degradation of A3G is not markedly affected by HBx. (**A**) Lack of effects of HBx on A3G ubiquitination as detected using immunoprecipitation. Huh-7 cells transfected with indicated plasmids were treated with MG-132 and subjected to cell lysis and purification of His-tagged A3G using Ni^2+^-NTA matrix under denaturing condition. Enriched His-tagged A3G was detected using anti-A3G and A3G modified by HA-tagged ubiquitin (HA-Ub) was detected using anti-HA antibodies. A3G and HBx in 1/10 input were also detected. (**B** and **C**) Treatment with proteasome inhibitor MG-132 or lysosome inhibitor leupeptin had no marked effect on A3G protein levels, with or without HBx. Huh-7 cells transfected and treated as indicated were lysed and subjected to immunoblot analysis.

Protein degradation via proteasome and lysosome pathways are key post-translational mechanisms that affect and determine target protein level at steady state. In order to explore the underlying mechanisms of HBx-mediated downregulation of A3G protein level, possible effects of HBx on A3G protein degradation were investigated. First, His-tagged A3G was co-transfected with HA-tagged ubiquitin, with or without co-transfected HBx. Transfected cells were treated with proteasome inhibitor MG-132 to block degradation of ubiquitinated proteins and A3G ubiquitination was analyzed by purification of His-tagged A3G followed by immunoblot using HA antibody. As shown in Fig. 4A, HBx co-expression resulted in decrease of A3G protein level without remarkably increasing ubiquitination of A3G. Furthermore, treatment of transfected cells with MG-132 had no obvious effect on A3G protein level, and HBx-mediated reduction of A3G was not affected by MG-132 treatment (Fig. 4B). These data suggest that proteasome pathway is not likely to be a major determinant of A3G protein level and HBx does not decrease A3G level by targeting more A3G for ubiquitination and proteasomal digestion.

Transfected cells were also treated with lysosome inhibitor leupeptin. Similar to MG-132, no notable effect on A3G protein level was observed with leupeptin treatment, and HBx-induced decrease of A3G was not affected by leupeptin either (Fig. 4C). Apparently, similar to proteasome degradation, lysosome-mediated protein degradation plays no significant role in either A3G protein level or its downregulation by HBx.

### Intracellular APOBEC3G protein is exported via exosome secretion

**Figure 5:**
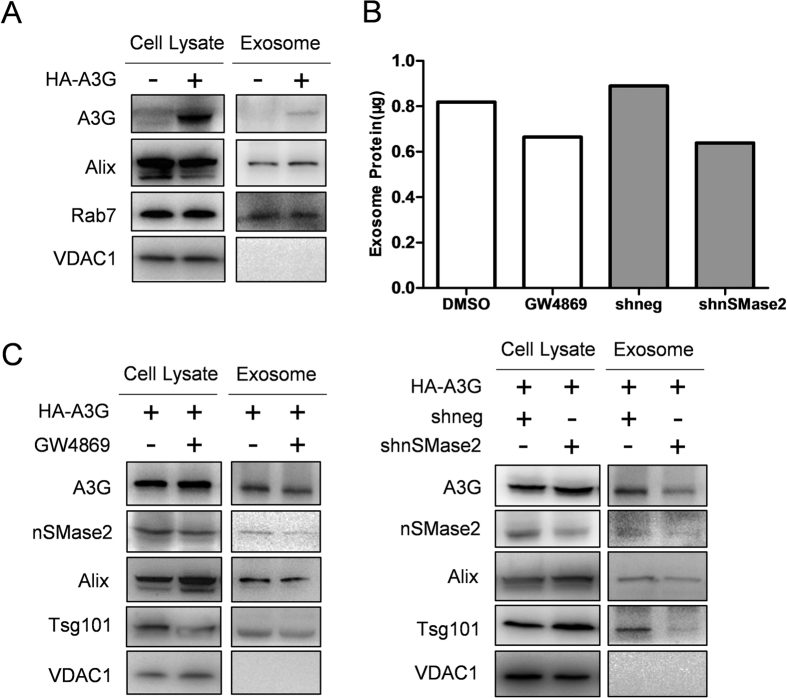
Intracellular A3G is exported via exosome secretion. (**A**) Detection of A3G protein in cell lysates and exosomes prepared from Huh-7 cells transfected with A3G expression plasmid or vector control. Exosome markers Alix and Rab7, and mitochondria marker VDAC1 were detected in parallel. (**B**) Treatment with GW4869 or transfection of plasmid expressing nSMase2-targeting shRNA (shnSMase2) inhibited exosome secretion by Huh-7 cells. Exosomes were prepared from 1E7 cells treated as indicated and total protein contents were quantified using BCA assay. DMSO vehicle and unrelated shRNA construct (shneg) were used as controls. (**C**) Treatment with GW4869 or shnSMase2 inhibited A3G export via exosome secretion and increased intracellular A3G protein level. Huh-7 cells transfected with A3G expression plasmid or vector control and treated as indicated were used for lysate and exosome preparation. In addition to A3G and exosome/mitochondria markers, nSMase2 was also detected to demonstrate knock-down by shnSMase2.

Since HBx decreases A3G protein level post-translationally, but without involving the two major protein degradation pathways, enhanced exocytosis was one of the remaining major possibilities that could be envisioned. In order to test such a hypothesis, whether A3G, a cytoplasmic protein that is not secreted, could be exported via the exosome production and secretion pathway was tested. Indeed, A3G protein was detected in exosomes purified from culture supernatants of Huh-7 cells transfected with A3G expression plasmid (Fig. 5A). Furthermore, when such cells were treated with nSMase2 inhibitor GW4869 or transfected with plasmid expressing shRNA targeting nSMase2 to inhibit exosome biogenesis and secretion^25^ (Fig. 5B), A3G protein level in exosomes slightly decreased whereas intracellular A3G slightly increased (Fig. 5C). These data indicated that not only is A3G exported out of the cell via exosome secretion, but more importantly, such externalization via exosomes contributes towards determining intracellular A3G protein level.

### HBx decreases intracellular APOBEC3G protein level through enhancing its export via exosomes

**Figure 6:**
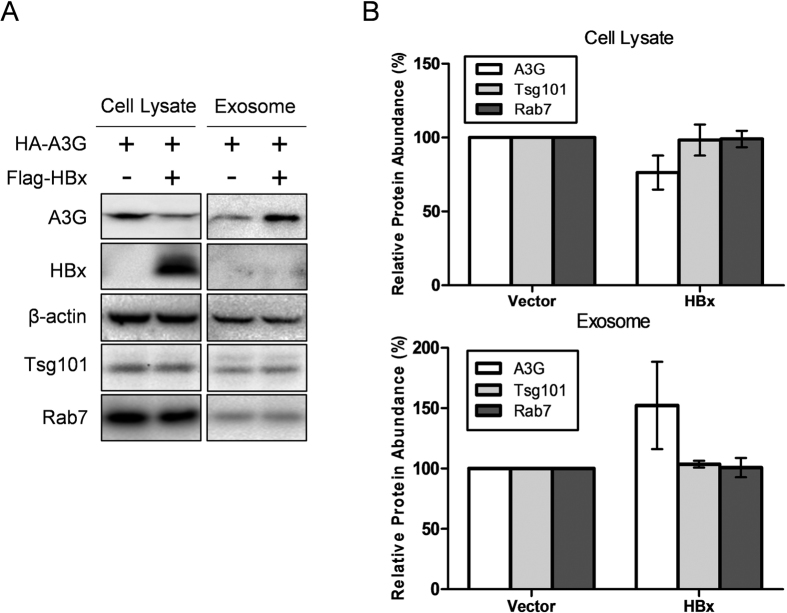
HBx increases export of A3G via exosome secretion. (**A**) Effects of HBx on A3G protein levels in cell lysates and exosomes. Huh-7 cells were transfected with indicated plasmids and 48 hours post transfection, cell lysates and exosomes were prepared and analyzed in immunoblot for A3G and HBx levels as well as exosome markers Tsg101 and Rab7. β-actin was used as loading control. (**B**) Relative quantities of A3G and exosome markers Tsg101 and Rab7 in cell lysates and exosomes as shown in (**A**) were respectively estimated using densitometry scanning taking values from vector-transfected cells as 1 (100%). Means and SD calculated from 3 independent experiments are shown.

Export of intracellular A3G via exosomes in cells without HBx expression and HBx-mediated post-translational and degradation-independent reduction of A3G protein levels suggest the possibility that HBx exerts its effects on intracellular A3G level by affecting its export through exosome production and secretion. When cell lysates and purified exosomes from cells transfected with A3G with or without HBx co-transfection were analyzed, increased A3G protein level was observed in exosomes prepared from A3G and HBx co-transfected cells compared to cells transfected with A3G alone (Fig. 6A,B). Correspondingly, intracellular A3G protein level was lower in cells co-transfected with HBx (Fig. 6A), reproducing results from earlier sections. No such changes could be observed for exosome markers Tsg101 and Rab7: their protein levels in cell lysates and exosomes did not change remarkably with or without HBx expression (Fig. 6A,B). These results showed that HBx selectively and specifically enhances A3G export through exosome secretion pathway, and this effect is, at least partially, responsible for HBx-mediated downregulation of intracellular A3G protein level.

## Discussion

A3G-mediated inhibition of HBV *in vitro* was first described^24^ very shortly after the discovery of the interplay between HIV-1 vif protein and A3G^26^. At the time, data on the actual role of HBx in HBV life cycle were still scarce and there was suspicion that HBx could be a possible functional homologue of vif that antagonizes A3G restriction^17^. However, in contrast to vif, which was shown to be essential for HIV-1 replication in A3G-expressing T cell lines^26^, HBx-null HBV genome is still capable of initiating considerable viral replication in the presence of overexpressed A3G^17^, as is also demonstrated in Supplementary Fig. S1. This was interpreted as evidence that HBx does not function as an essential defense against A3G restriction as vif does and analogy between HBx and vif was no longer given much attention. In this work, we present results demonstrating that similar to vif 26,27, HBx selectively, dose-dependently and post-translationally reduces intracellular protein level of A3G *in vitro* (Figs 1, 2 and 3), yet unlike vif, HBx-mediated A3G downregulation appears to be minimally associated with A3G ubiquitination or degradation (Fig. 4), but involves enhanced A3G externalization via exosome secretion instead (Figs 5 and 6). These data suggest the possibility that HBx might after all act as a functional homologue of vif to counteract A3G-mediated inhibition, although the underlying mechanisms could be significantly dissimilar.

A3G is constitutively expressed in HIV-1 target CD4^+^ T cells as well as many other hematopoietic cell types, and higher protein levels can be induced by interferon-α treatment in macrophages and dendritic cells^28^,^29^. Many studies have demonstrated constitutive and induced A3G expression in primary hepatocytes and hepatoma cell lines at mRNA level^29^,^30^,^31^,^32^,^33^, but data on protein level expression are fairly scarce^29^,^32^,^33^. We also failed to detect endogenous A3G protein in cultured Huh-7 cells using commercial antibodies (Fig. 5A), and characterized HBx’s effects on A3G using exogenous protein expressed from transfected plasmid as has been the common approach adopted by most studies of A3G performed in liver cells^17^,^18^,^19^,^24^,^30^,^34^. Therefore, the question whether HBx could decrease the protein level of endogenous A3G, constitutive and/or induced, in hepatocytes in a similar fashion as it does that of exogenous A3G in transfected hepatoma cells (Figs 1 and 2), remains to be addressed by further studies both *in vitro* and *in vivo*.

Previously, besides transcriptional upregulation stimulated by treatment with cytokines such as interferon-α, the only other well characterized mechanism of A3G regulation has been vif-mediated downregulation through enhanced ubiquitination and proteasomal degradation. Results presented in this work suggest the existence of another potential mechanism of regulating intracellular A3G activity: altered A3G externalization through exosome secretion (Figs 5 and 6). Exosomes are known to contain lipids, proteins and nucleic acids derived from and characteristic of the producing cells^23^. Although exosome studies in recent years have tended to focus on its functions as a device for intercellular transport and communication, exosome secretion was initially discovered and characterized as a process of exporting and thereby reducing obsolete plasma membrane proteins during erythrocyte maturation^35^,^36^,^37^. Later, release of cytosolic β-catenin via exosomes was also demonstrated and shown to be a process that antagonizes Wnt signaling^38^. It is therefore not without precedence that intracellular level of a cytosolic protein like A3G can be regulated by exosome-mediated externalization. However, there does not seem to exist any prior report of a pathogen interfering with such a process to antagonize a cellular restriction factor. It is possible that further work might identify comparable cases in other host-pathogen interaction contexts. Meanwhile, exosomes could end up being taken up by other cells and deliver its contents to the latter, and there have been results showing that exosome-mediated cell-to-cell transmission of interferon-induced proteins provides recipient cells with antiviral resistance^39^. In this light, although HBx-induced higher export of A3G via exosomes might relieve A3G-mediated restriction in HBx-expressing cells, whether exported A3G contained in exosomes has any effects on anti-HBV defense of recipient cells is also an interesting question worth pursuing. Since A3G is capable of inhibiting viruses other than HBV, most notably HIV-1, the possibility that HBx-mediated A3G export via exosomes affects the innate immunity against other viral infections in recipient cells in co-infections is worth exploring too.

It is unclear how HBx actually facilitates the externalization of A3G via exosomes, but since the effect is specific for A3G but no other tested exosome proteins (Fig. 6), generalized upregulation of exosome biogenesis could be ruled out. As HBx itself is not detectable in exosomes (Fig. 6), co-export through direct or indirect interactions between HBx and A3G is also not very likely. Although morphogenesis of HBV progeny virion has been shown to overlap significantly with exosome biogenesis pathway^40^,^41^, our results indicate that HBx alone could promote A3G export without involving other viral proteins or processes. Additional in-depth studies are clearly required to elucidate the underlying molecular details.

In summary, data presented in this work suggest a possible new mechanism of HBx-mediated HBV activation: through antagonizing the host restriction factor APOBEC3G. Proving the functional relevance of HBx-mediated decrease of A3G protein level in HBV life cycle is complicated (Supplementary Fig. S1), because HBx has been shown to promote HBV replication through multiple mechanisms^11^ that are possibly but not necessarily A3G-independent. Conclusive evidences would only become obtainable when HBx mutants that lose HBV-activating capability but retain A3G-decreasing capability, or other agents that specifically enhance A3G export, can be identified in future. To further complicate the issue, whether other HBV proteins exert any direct or indirect effects on A3G is currently unknown. It is not impossible that externalization of A3G promoted by HBx could be reinforced or counteracted by other HBV components through similar or dissimilar mechanisms. Nevertheless, our preliminary results show that intracellular levels of transfected A3G were lower in cells stably transfected with HBV genomes compared to control (Supplementary Fig. S2). Therefore, HBV expression and replication is indeed associated with lowered A3G protein level *in vitro*, which could be at least partially attributable to HBx (Figs 1–6). Hopefully, this work will pave the way for future efforts along this line that eventually lead to a better and more thorough understanding of the complex interplay between HBV and A3G.

## Methods

### Plasmids, cell culture and transfection

HBx (GenBank Accession Number AAD16255.1), A3A (AKE33285.1), A3C (AAH11739.1), A3F (AAH38808.1), A3G (AAH61914.1), A3H (AAH69023.1) coding sequences were amplified by PCR and cloned downstream of CMV promoter into pcDNA3 vector (Invitrogen). Flag or HA tags were introduced in some constructs to enable easier detection of expressed protein. Plasmid carrying terminally redundant 1.1X copy HBV genome downstream of CMV promoter with the HBx ORF nulled by introducing premature termination at residue 8 (1.1HBV-X^−^) has been previously described^10^. CMV-driven eGFP expression plasmid (Beyotime) was used for transfection efficiency control. HA-tagged ubiquitin (HA-Ub) expression plasmid was a gift from Prof. Mujun Zhao of Shanghai Institute of Biochemistry and Cell Biology, Chinese Academy of Scienes.

Sequences encoding short hairpin RNA that targets nSMase2 (shnSMase2) were chemically synthesized and inserted into pLKO.1-eGFP vector under the control of U6 promoter. shnSMase2 target sequences are 5′-AATGCTACTGGCTGGTGGACC-3′ as previously reported^42^. Control shRNA (shneg) expressing plasmid was also constructed with unrelated target sequences 5′-CCTAAGGTTAAGTCGCCCTCG-3′ bearing minimal homology to known human genes.

Huh-7 cells were maintained in Dulbecco’s Modified Eagle’s Medium supplemented with 10% fetal bovine serum, 100 U/ml penicillin G/streptomycin sulfate and 10 mM HEPES (all from Invitrogen) at 37 °C and 5% CO_2_. Transfections were performed using Turbofect (Thermo Scientific) according to manufacturer’s instructions and 24 hours later, cells were changed into fresh media. Transfected cells and culture supernatants were collected at 48 hours post transfection for cell lysate and exosome preparation, respectively.

Cycloheximide treatment was performed by adding cycloheximide (Ameresco) dissolved in DMSO (Sigma) to a final concentration of 100 μg/ml at 36 hours post transfection. Treated cells were collected at different time points and saved for subsequent lysate preparation and immunoblot analysis.

### Antibodies

A3G (60100-1) and Rab7 (55469-1-AP) antibodies were purchased from Proteintech. Antibodies against Alix (A2215), TSG101 (A2216), and VDAC1 (A0810) were purchased from ABclonal. Peroxidase-labeled β-actin antibody (A3854) and Flag antibody (F3165) were purchased from Sigma. nSMase2 (sc-166637) antibody was purchased from Santa Cruz Biotechnology. HA antibody (M20003) and eGFP antibody (M20004) were purchased from Abmart. Mouse monoclonal HBx antibody was custom made as previously described^43^.

### Western and Southern blot

Transfected cells were harvested using a scraper and washed three times in cold phosphate buffered saline (PBS, pH 7.3). Pelleted cells were then lysed in RIPA buffer containing protease inhibitor cocktail (Merck), and lysates were subjected to conventional SDS-PAGE and immunoblot analyses. Blots were developed using chemiluminescent substrate (Millipore) and captured using ChemiDoc XRS+ (BioRad).

Cells transfected with HBV genome plasmid were subjected to extraction of intracellular capsid-associated HBV DNA followed by agarose electrophoresis and Southern blot using ^32^P-labelled HBV probes, as previously described^44^. Blots were exposed to phosphorimage plates and scanned using Typhoon FLA9500 (GE Health).

### Ubiquitination analysis

Huh-7 cells were transfected with HA-Ub plus His-A3G, Flag-HBx or corresponding control plasmids and 2 days later, cells were treated with 40 μmol/L MG-132 (Sigma) for 6 hours before harvest to inhibit protein degradation in proteasomes. Scraped and washed cells were lysed in denaturing lysis buffer (6 mol/L guanidinium-HCl, 0.1 mol/L Na_2_HPO_4_/NaH_2_PO_4_, 10 mmol/L Tris-HCl pH 8.0, with 5 mmol/L imidazole and 10 mmol/L β-mercaptoethanol). Lysates were cleared by centrifugation and after saving 1/10 portions as inputs, His-A3G was purified using Ni^2+^-NTA agarose beads (QIAGEN) by incubation with shaking at 4 °C for overnight. The beads were pelleted and washed sequentially with lysis buffer and wash buffer (lysis buffer with guanidinium-HCl replaced by 8 mmol/L urea), and bound His-A3G was eluted using elution buffer (0.2 mmol/L imidazole, 0.15 mmol/L Tris-HCl pH 6.8, 0.72 mmol/L β-mercaptoethanol, 5% SDS, 30% glycerol). Eluents were subjected to immunoblot using HA and A3G antibodies, and saved inputs were analyzed in parallel using A3G and HBx antibodies.

### Exosome preparation

Exosomes were prepared from cell culture supernatants using the classical method of differential centrifugation essentially as previously reported^10^. Briefly, supernatants were first centrifuged at 300 *g* for 10 min to remove detached cells and cell debris was removed by centrifugation at 2,000 *g* for 15 min followed by centrifugation at 10,000 *g* for 30 min. The clarified supernatant was filtered through a 0.22 μm filter and ultracentrifuged at 100,000 *g* for 90 min using an SW28 rotor (Beckman) to pellet exosomes. Exosomes were washed by resuspension in 10 ml PBS and ultracentrifugation for 90 min at 100,000 *g* using a SW41 rotor (Beckman). Pelleted exosomes were resuspended in PBS and measured for total protein quantities using BCA method (BioRad). All the above procedures were performed at 4 °C and purified exosomes were stored at −80 °C before use.

### Data processing and analysis

Densitometry scanning was performed on Western and Southern blot images using MultiGauge V2.2 software (Fujifilm). When necessary, results from 3 independent biological replications of the same experiment were used to calculate means and standard deviations, which were then used for plotting.

## Additional Information

**How to cite this article**: Chen, R. *et al*. Hepatitis B virus X protein is capable of down-regulating protein level of host antiviral protein APOBEC3G. *Sci. Rep.* **7**, 40783; doi: 10.1038/srep40783 (2017).

**Publisher's note:** Springer Nature remains neutral with regard to jurisdictional claims in published maps and institutional affiliations.

## Supplementary Material

Supplementary Information
