# Supplementary material for: Hepatitis B virus X protein is capable of down-regulating protein level of host antiviral protein APOBEC3G
Source: Sci Rep. 2017 Jan 18;7:40783. doi: 10.1038/srep40783 (PMC5241686; doi:10.1038/srep40783)
Supplement: Supplementary Information [file srep40783-s1.pdf]

Title: Hepatitis B virus X protein is capable of down-regulating protein level of host antiviral protein APOBEC3G

Authors: Ruidong Chen, Xue Zhao, Yongxiang Wang, Youhua Xie, and Jing Liu

Supplementary Information

Figure S1. HBx-mediated decrease of A3G protein level correlates with elevated HBV replication.

Figure S2. Analysis of protein levels of exogenously expressed A3G in HepG2 cells stably transfected with HBV genome.

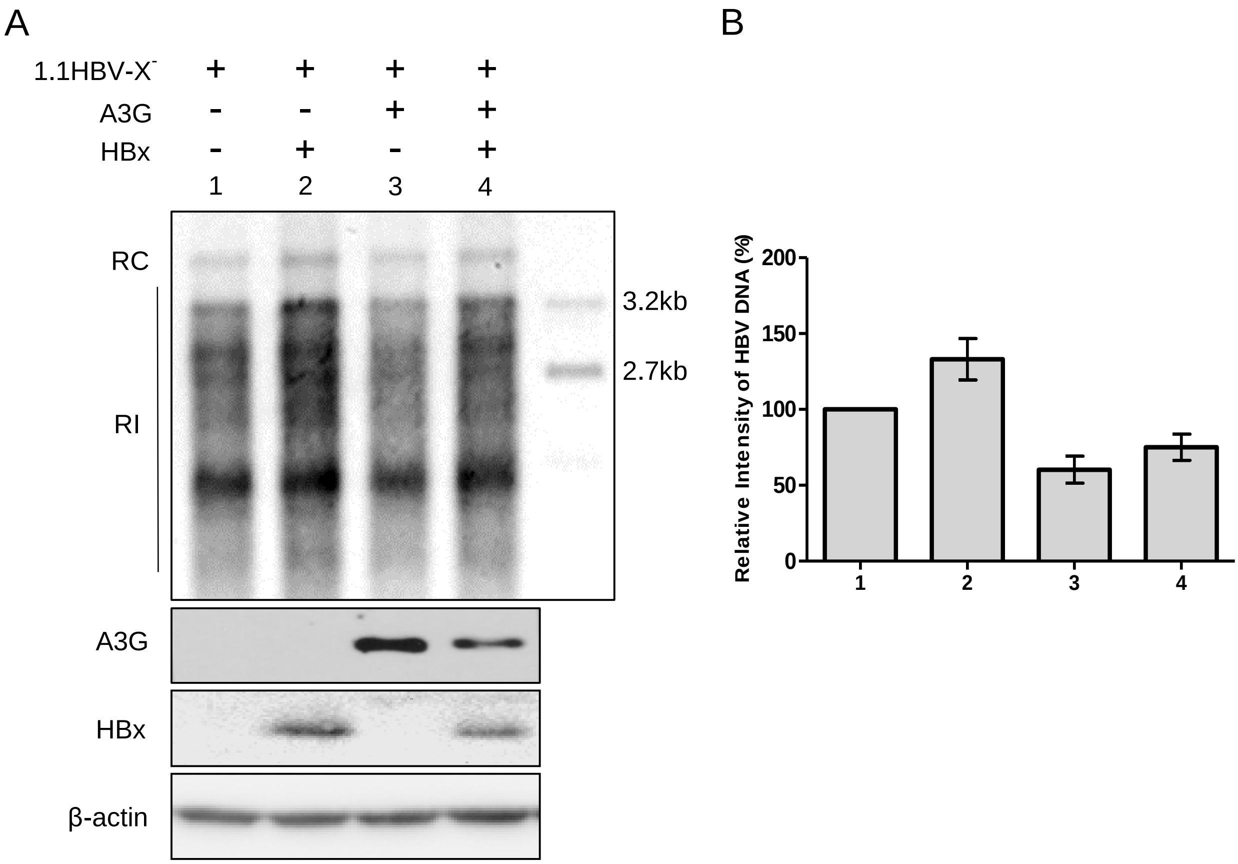

Supplementary Figure S1. HBx-mediated decrease of A3G protein level correlates with elevated HBV replication.

(A) Effects of A3G and HBx on replication of HBV genome carrying nulled HBx ORF (HBV-X$^-$). Huh-7 cells were transfected with indicated plasmids and at 48 hours post transfection, cells were harvested and a portion was used for analyzing A3G and HBx levels in immunoblot. Intracellular capsid-associated HBV DNA was extracted from remaining cells and analyzed in Southern blot using HBV-specific probe. Positions of mature relaxed circular (RC) progeny viral DNA and replication intermediates (RI) are indicated. β-actin was used as loading control. **(B)** Relative quantities of RC plus RI HBV DNA as shown in (A) were estimated using densitometry scanning taking the first lane (single transfection with 1.1HBV-X$^-$) as 1 (100%). Means and SD calculated from 3 independent experiments are shown.

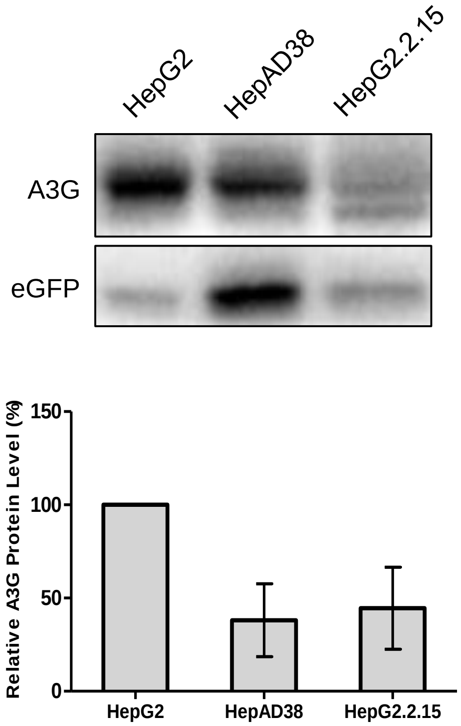

Supplementary Figure S2. Analysis of protein levels of exogenously expressed A3G in HepG2 cells stably transfected with HBV genome. Normal HepG2 and two HepG2-derived cell lines stably transfected with constitutively (HepG2.2.15) or inducibly (HepAD38) expressed HBV genome were co-transfected with plasmids encoding A3G and eGFP. Protein levels in cell lysates wereanalyzed using immunoblot and relative quantities of A3G were estimated using densitometry scanning taking values from normal HepG2 cells as 1 (100%). Means and SD calculated from 3 independent experiments are shown.
